# Understanding the microbial fibre degrading communities & processes in the equine gut

**DOI:** 10.1186/s42523-022-00224-6

**Published:** 2023-01-12

**Authors:** Georgia Wunderlich, Michelle Bull, Tom Ross, Michael Rose, Belinda Chapman

**Affiliations:** 1 Tasmanian Institute of Agriculture, University of Tasmania, Hobart, Australia; 2 Quantal Bioscience Pty Ltd, Castle Hill, Australia

**Keywords:** Equine, Microbiome, Gastrointestinal tract, Fibre, CAZyme, Anaerobic fungi, Health

## Abstract

The equine gastrointestinal tract is a self-sufficient fermentation system, housing a complex microbial consortium that acts synergistically and independently to break down complex lignocellulolytic material that enters the equine gut. Despite being strict herbivores, equids such as horses and zebras lack the diversity of enzymes needed to completely break down plant tissue, instead relying on their resident microbes to carry out fibrolysis to yield vital energy sources such as short chain fatty acids. The bulk of equine digestion occurs in the large intestine, where digesta is fermented for 36–48 h through the synergistic activities of bacteria, fungi, and methanogenic archaea. Anaerobic gut dwelling bacteria and fungi break down complex plant polysaccharides through combined mechanical and enzymatic strategies, and notably possess some of the greatest diversity and repertoire of carbohydrate active enzymes among characterized microbes. In addition to the production of enzymes, some equid-isolated anaerobic fungi and bacteria have been shown to possess cellulosomes, powerful multi-enzyme complexes that further enhance break down. The activities of both anaerobic fungi and bacteria are further facilitated by facultatively aerobic yeasts and methanogenic archaea, who maintain an optimal environment for fibrolytic organisms, ultimately leading to increased fibrolytic microbial counts and heightened enzymatic activity. The unique interactions within the equine gut as well as the novel species and powerful mechanisms employed by these microbes makes the equine gut a valuable ecosystem to study fibrolytic functions within complex communities. This review outlines the primary taxa involved in fibre break down within the equine gut and further illuminates the enzymatic strategies and metabolic pathways used by these microbes. We discuss current methods used in analysing fibrolytic functions in complex microbial communities and propose a shift towards the development of functional assays to deepen our understanding of this unique ecosystem.

## Introduction

The equine hindgut is a complex naturally occurring fermentation system of powerful lignocellulolytic microbes. ‘Fibre’ (complex plant polysaccharides) degradation is an essential process in equine digestion, allowing the liberation of vital energy sources. Despite consuming a strictly plant based diet, equids lack the diversity of enzymes needed to break down complex plant polymers alone and have evolved symbiotic relationships with resident gut microorganisms to produce the enzymes that facilitate plant matter degradation [1]. These microbes ferment complex carbohydrates in plant material into short chain fatty acids, contributing 60–70% of the horse’s daily energy requirements [2].

An understanding of the equine hindgut microbial ecosystem and the parameters that control and affect it can greatly facilitate our understanding of host-microbe interactions and how we can optimise the functionality of resident microbes and ultimately maximise energy yield within this environment. Our understanding of the role of bacteria in hindgut fermentation is progressing [3–8], however, knowledge of the function of other microbial communities, including archaea and fungi, in the hindgut is still lagging [9]. This review outlines the fibrolytic microbial community composition of the equine hindgut and elucidates the enzymatic processes and metabolic pathways that allow the break down of complex polysaccharides, as well as detailing current and future techniques to assist further understanding of the mechanisms and functional genes underlining these processes.

## The equine digestive habitat

### Anatomy of the equine gastrointestinal tract

The majority of studies undertaken on herbivore digestive systems, and particularly the microbial communities residing in them, have been done in ruminants, specifically bovine and ovine. The horse gut shares several similarities with the ruminant gut, having combined caecal and colonic regions, with both being heavily dependent on their gut microbiota for digestion and nutrition [10]. Horses are monogastric herbivores, however, and do not regurgitate digesta for further break down like foregut fermenting ruminants, having a greater dependence on resident hindgut microbes for fermentation and digestion [11]

The equine gastrointestinal tract (GIT) is aerobic to anaerobic from anterior to posterior, due to the intake of oxygen with feeding and the subsequent utilization of most of this oxygen by aerobic fermenters prior to reaching the hindgut [5]. The horse’s stomach is the smallest of any livestock or domestic animal relative to its size, having a capacity of only 9–18 L [12]. Most food degradation takes place in the large intestine which makes up over 60% of the equine GIT. Upon entry to the large intestine, 85–95% of cell wall derived carbohydrates are undigested [12]. Digesta enters the large intestine approximately 3 h after feeding and is fermented for 36–48 h in the caecum [11]. The caecum sits at a pH of 6.3–7.5, ideal for the growth of a plethora of anaerobic bacteria, fungi and protozoa [11]. Additionally, neurological signalling during feeding times trigger the caecum to increase in capacity and mobility to enhance microbial-digesta interactions, allowing microbes to efficiently degrade difficult plant biomass and subsequently synthesise vital energy sources for the animal [11].

### Equine gut microbiome

The dependence of equids on their gut microbiota and the consequent importance of this population has led to the viewing of the host and its microbes as one unit when evaluating health and host phenotype, referred to as the ‘holobiont’ (a host and its microbiota) [13]. Microbial colonization of the equine gut is generally through diet, supplements, coprophagy (faecal consumption) or other methods of ingestion [14–16]. Studies on the meconium (first defecation) of newborn foals have found that their early GIT microbiome largely reflects bacteria found in the maternal milk [17] with other studies showing the equine microbiome generally begins to stabilise between one and two months of age [18]. A database has recently been compiled to analyse taxon-associated host phenotypes and is available at http://addagma.omicsbio.info/ to allow users to make biologically relevant queries about microbial related trends in equine health and disease [19]. The database summarises experimental observations found between domestic animals and their gut microbiota, however also highlights the lack of studies assessing these relationships in equids, with studies related to horse microbial phenotypes being significantly fewer than those of cattle, pigs and chickens [19]. The entire equine GIT can contain up to 10^15^ bacterial cells [9], and studies have reported as many as 10^4^ fungal zoospores/mL of caecal content [11], although these numbers do not necessarily equate to their functional value in the gut. The most important end product of fermentative processes carried out by these microbes are volatile fatty acids, such as propionate, acetate and butyrate [20] which are vital energy sources for horses, as well as carbon dioxide, water, methane, vitamins and several amino acids [21].

Fibre degradation in the equine gut is primarily carried out by anaerobic bacteria and fungi, facilitated by facultatively aerobic yeasts and methanogenic archaea [22]. For many years protozoa were additionally considered contributors to fibre degradation in the equine gut due to their ability to rapidly adhere to and colonize plant tissue [23, 24]. An older study [25] showed their potential to enhance bacterial degradation of pectin and hemicelluloses, particularly arabinogalactan and galactomannan components [25]. However a later investigation found little impact of these microbes on plant digestion and fibrolysis, and concluded that they have a minimal role in directly degrading plant matter [9]. Bacteriophages have also been suggested to influence the fitness of intestinal cellulolytic bacteria and support colonisation, although do not have a direct role in fibre break down [26].

### Diet

**Fig. 1:**
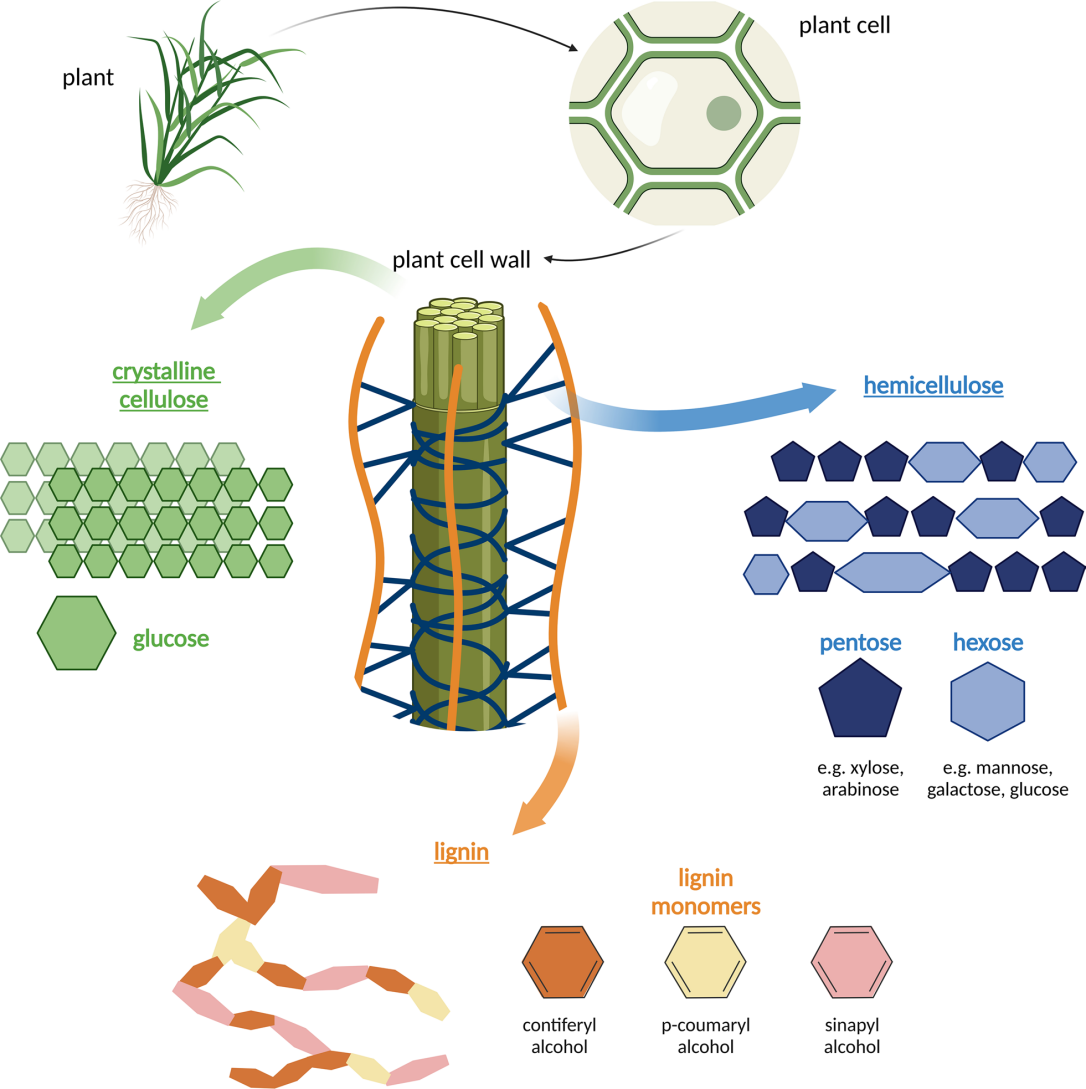
Structure of the main components of plant biomass (cellulose, hemicellulose, and lignin). All components contain amorphous areas and variable structures and will not always present as the structures depicted above. (Adapted from [28]). Figure made in BioRender

Being herbivores, equids source their nutrition from plants, either through wild forages or commercial feeds. Plant cell walls are composed of lignocellulose, a structure made of two carbohydrate polymers, cellulose and hemicellulose, and an aromatic heteropolymer, lignin, to bind the polysaccharides together (relative abundances of approximately 45%, 30% and 25% respectively) (see Fig. 1) [27].

Cellulose, the major component of plant biomass, is one of the most abundant polysaccharides on earth [29]. Cellulose is composed of closely stacked disaccharide cellobiose fibres linked by β (1–4)-glycosidic bonds, organised in crystalline structures with scattered amorphous areas. Despite being a relatively simple compound, the proximity of these fibres to each other makes accessing the bio-nutrients of cellulose difficult. Hemicellulose molecules are the second most abundant plant polysaccharide and present as polymers of pentoses and/or hexoses, interconnected by covalent hydrogen bonds [28]. The main structures of hemicellulose are xylan, xyloglucan, and galactomannan which vary in their structure and makeup depending on their respective sugars. Sugars in hemicellulose are linked similarly to cellobiose fibres by β (1–4)-glycosidic bonds and, occasionally, β (1–3)-glycosidic bonds [30]. Finally, lignin is an amorphous heterogenous polymer composed of three aromatic alcohols [28]. The quantity and distribution of these aromatic alcohols varies between plant species. Lignin is associated with hemicellulose and cellulose by ester and hydrogen bonds. It is these bonds between the different structural components of plant biomass, as well as the rigidity and complexity of individual components that create the overall resistance to degradation of lignocellulose complexes.

Unsurprisingly, the composition of plant structure and contribution to herbivore feed has had a demonstrated effect on the microbiome composition and overall fibrolytic capacity of the equine gut [14, 31–34]. Forages available for grazing equids change substantially across the globe and throughout the year, leading to corresponding changes in feed nutrient value and digestibility. Generally speaking, grasses grown during a rainy season are higher quality and more nutrient dense, while a dry season brings increased lignin in plants, the least digestible fibre component of plants, and decreased nitrogenous content [35]. Feeds harvested during dry climatic conditions generally lead to reduced fibre utilization due to the low nitrogenous content of this feed meaning there are less nitrogenous precursors for the synthesis of microbial compounds such as enzymes [35]. The effects of this may be partially overcome with nitrogenous dietary supplementation which has been shown to stimulate gut fibrolytic activity and increase degradation of low quality fibre [35].

Julliand and Grimm [14] provide an overview on the impact of diet on the equine hindgut microbiome. Generally, studies show diets high in starch have adverse effects on microbial populations in the equine gut, reducing cellulolytic bacterial counts and overall fibrolytic capacity [34, 36, 37]. Such feed types are common among domesticated pet horses in the form of pelleted horse feeds high in barley, oats and corn [37]. Correspondingly, these high concentrate diets can lead to an increase in amylolytic bacteria, the primary one being *Lactobacillus* species [38], overpopulation of which can lead to gut acidosis and ultimately colitis and laminitis [3, 39, 40]. High fibre diets, in contrast, seem to maximise the fibrolytic capacity of the equine gut, leading to increased cellulolytic and xylanolytic bacterial counts [32, 41], as well as decreased concentrations of bacteria typically associated with laminitis induction, such as *Lactobacillus* [4]. While these studies highlight apparent trends between forage composition and microbial populations, other studies have suggested the reaction of the horse gut microbiome to changes in plant structure to be largely individualised between horses, with implications on metabolic health remaining to be elucidated [42].

### Fibre degrading enzymes

The complete break down of plant biomass requires the presence of multiple enzymes to target different components of lignocellulose. Carbohydrate degradation involves two steps, the first being hydrolysis of plant polysaccharides, and the second being the fermentation of the resulting simple sugars into short chain fatty acids [14].

‘CAZymes’ are carbohydrate active enzymes which catalyze the break down and assembly of glycoconjungates and glycans [43]. Currently there are six main CAZyme classes, glycoside hydrolases (GHs), glycosyl transferases (GTs), polysaccharide lyases (PLs), carbohydrate esterases (CEs), carbohydrate binding modules (CBM) and auxiliary activities (AAs) which are redox enzymes that facilitate CAZyme function. These enzymes are classified according to the different mechanisms in which they break down substrates, as well as their three-dimensional folding structure characteristics and protein sequence similarities [44]. An overview of different CAZymes is available at http://www.cazy.org/ which has recently been updated and reviewed [45].

Endoglucanases, exoglucanases and β-glucosidases are the three main cellulase groups that act synergistically and simultaneously to break down cellulose using hydrolysis [46]. Endoglucanases target amorphous areas of the crystalline cellulose matrix and cut into them, producing a chain end that exoglucanases are then able to bind to and cleave, releasing the cellobiose fibres and individual glucose monomers [46]. Finally, the cellobiose is degraded to glucose monomers by β-glucosidases [46]. While hydrolysing cellulases are the prominent degraders of cellulose, other cellulases exist which utilize other modes of action, such as cellodextrinases and cellobiose phosphorylases, which use phosphorylation-mediated cleavage, or oxidoreductases which use an oxidative mode of action. Various microorganisms can produce cellulases, including bacteria, fungi, metazoan and some animals like termites, snails and crayfish [47–49].

Several enzymes are involved in the break down of hemicellulose which act specifically on the different glycol units and glycosidic bonds. These hemicellulases include endo- and exo-β-glucanases and xylanases, polygalacturonases, pectin methyl esterases, β-mannanases, feruloyl esterases, pectin and pectate lyases and arabinofuranosidases [50]. Hemicellulases are primarily produced by saprophytic microbes isolated from decaying plant and animal material [51].

Current research on lignin degradation has focused on the role of fungi in this process [52], however bacteria have also been demonstrated to produce enzymes enabling lignin break down [53]. The enzymes primarily responsible for lignin break down are laccases and peroxidases and can be generally divided into two main groups; lignin modifying enzymes and lignin degrading auxiliary enzymes, the latter of which are unable to degrade lignin on their own but are necessary to complete the degradation process. These proteins primarily fall under the ‘auxiliary activities’ family classification within the CAZy database. Lignin degradation is an oxidative process, therefore any microbes involved in this process within the equine gut are likely rare and difficult to detect due to the prominent anaerobicity of the equine hindgut [54]. Further culture-independent research is needed to further elucidate microbial methods of lignin break down within the equine GIT.

CAZymes are a growing focus in a range of research fields due to their diverse industrial applications and environmental distribution. Anaerobic gut dwelling bacteria and fungi possess some of the greatest diversity and repertoire of CAZymes amongst characterized microbes [55, 56], and the ecosystem of the equine may even serve as an environment for horizontal gene transfer of CAZymes between organisms, as demonstrated in ruminant species [57]. Comprehensive gene cataloguing of the faeces from eleven endurance trained horses found 137 different CAZymes through shotgun sequencing methods, the majority (85.4%) belonging to glycoside hydrolase and polysaccharide lyase families [58], highlighting the wealth of fibre degrading enzymes in the horse gut.

**Fig. 2:**
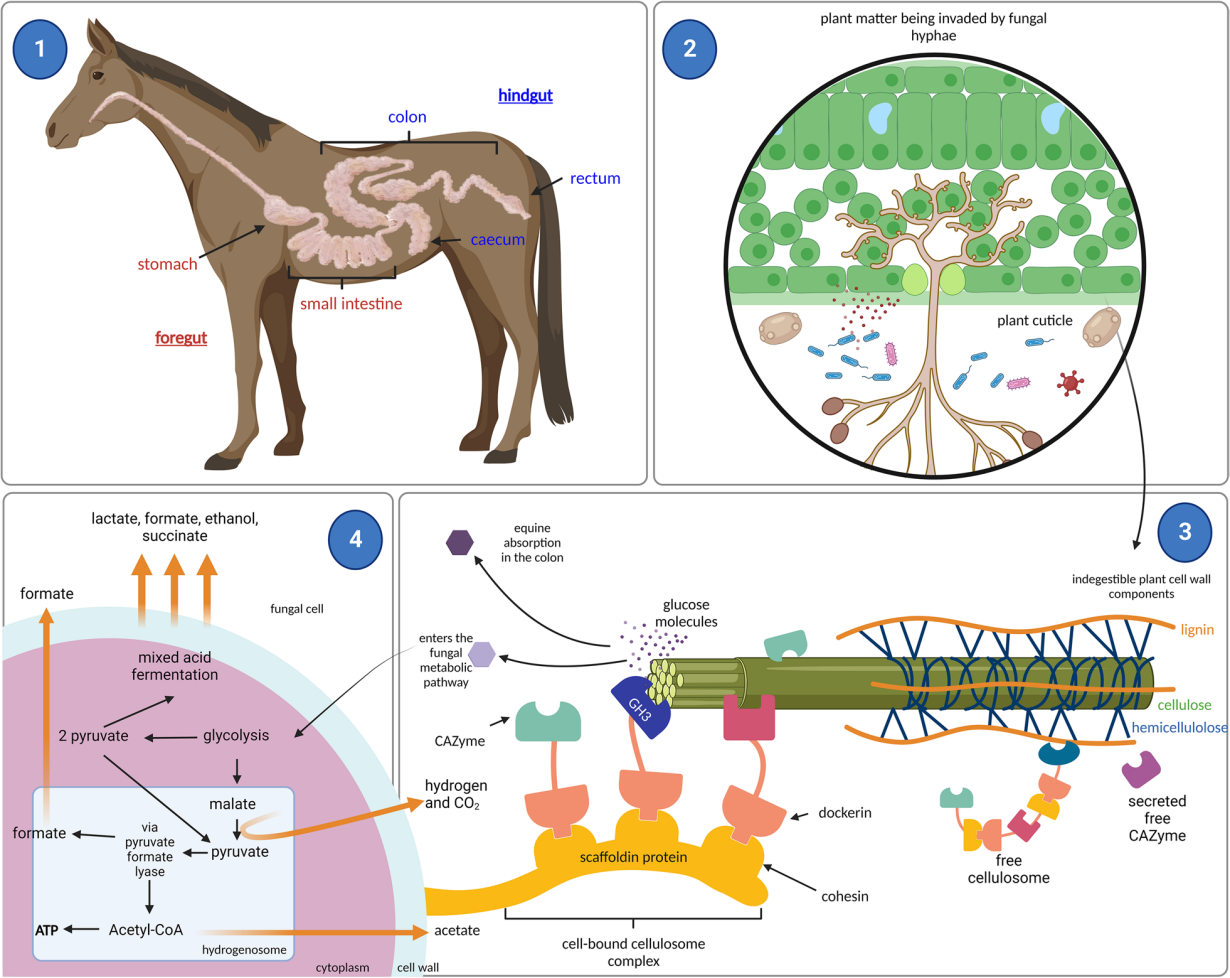
The role of fungi in plant break down and metabolism. **1** Schematic diagram of the equine digestive system (red text indicates the foregut, blue text indicates the hindgut). The majority of hindgut digestion occurs in the caecum. **2** Plant matter in the caecum is invaded by penetrative hyphae of anaerobic fungi. **3** Overview of the enzymatic activity of anaerobic fungi (adapted from [62]). Anaerobic fungi degrade plant biomass within the equine caecum through several enzymatic strategies; free carbohydrate active enzymes (CAZymes), cell bound cellulosome complexes and free cellulosomes secreted by the cell. Cell bound cellulosome example given is of glycoside hydrolase 3 which converts cellulose to monosaccharide glucose molecules via β-glucoside activity. These glucose molecules can then be absorbed in the equine gut or enter the fungal metabolic pathway. **4** Example of energy metabolism of *Piromyces* sp. E2 (adapted from [63]). Glucose molecules enter the glycolysis pathway, the product of which are two pyruvate molecules which either enter a mixed acid fermentation in the cytosol, or the hydrogenosome for ATP generation. Major by-products of fungal energy are indicated by the thick orange arrows. Figure made in BioRender

The equine hindgut also houses microorganisms that are exceptional because they can produce ‘cellulosomes’ which are multi-enzyme complexes made up of multiple CAZymes. Cellulosomes co-ordinate lignocellulolytic enzymes of similar functions to colocalise within the complex for enhanced degradation [59]*.* Cellulosomal enzymes are bound to a non-enzymatic membrane-anchoring protein, a scaffoldin, via a modular dockerin domain attached to the enzymes which interact and bind with cohesion proteins on the scaffoldin [60, 61], thus creating dynamic and powerful catabolic complexes (see Fig. 2).

## Fungi

### Anaerobic fungi

Anaerobic fungi are believed to be major contributors to fibrolysis and hindgut fermentation [22], found to represent at least 20% of microbial biomass in the rumen (63). Anaerobic fungi are significantly better degraders of plant cell walls compared to bacteria, due to their larger repertoire of fibre degrading enzymes and cellulosomes and their ability to mechanically invade plant tissue with penetrative hyphae [56, 62, 64]. Yet, this niche group of microorganisms remains greatly understudied within herbivore research. Neocallimastigomycota is the only described anaerobic fungi phylum and their production of lignocellulolytic enzymes are central to a range of agricultural, biogeochemical, and nutritional processes [62]. First identified in the herbivore system as zooflagellates, early research on anaerobic fungi was centralised around their role in breaking down feeds [65, 66]. These fungi have since been isolated from diverse and extreme environments, including in bedrock deep in the earths biosphere [67]. The uniqueness of this group of fungi stretches beyond their ability to survive under the anaerobic conditions of the equine gut, and has propelled research into understanding their taxonomy, enzymology, morphology, and diversity in host animals. Hess et al. provides a comprehensive overview of past, present and future research on these distinctive organisms [62].

All described anaerobic fungi are members of the phyla Neocallimastigomycota, which contains a single order (*Neocallimastigales)* and family (*Neocallimastigaceae)*. Within this family are 20 recognized genera, eleven of which have only been characterised in the last five years [68–71]. To date, at least six genera have been isolated from the equine gut (*Piromyces* [72–79], *Orpinomyces* [72, 80], *Neocallimastix* [72–74, 80], *Anaeromyces* [72, 74, 80], *Caecomyces* [72–74, 77] and *Khoyollomyces* [68, 73, 80]), with several more uncharacterised species also being found with the development of next generation sequencing technologies [73, 74, 80]. A list of publicly available whole genomes of anaerobic fungi was compiled by Hess et al. [62] and included one horse habituating organism, *Piromyces finnis* [81].

Anaerobic fungi obtain energy from breaking down plant carbohydrates using cellulolytic enzymes, prominently CAZymes. An overview of CAZymes found in well-characterized equine microbes are shown in Table 1. The functional diversity of CAZymes in gut dwelling fungi is impressive and far exceeds that of species currently used in cellulolytic cocktails for biotechnological purposes, such as *Aspergillus niger* and *Trichoderma resii* [59, 82]. In fact, early-branching anaerobic fungi encode the largest number of biomass-degrading enzyme genes found in nature to date, with the genome of a strong plant-degrading anaerobic fungus typically harbouring 200–300 CAZyme specific genes [44, 81]. This has been attributed to early horizontal gene transfer of bacterial hemicellulases to anaerobic fungi [81], providing anaerobic fungi a more diverse repertoire of enzymes compared to later diverging fungi which lack substrate catabolism diversity [56]. As such, anaerobic fungi have demonstrated efficient digestion of all major components of plant wall material including cellulose, xylan and galactomannan [83]. In ruminant GITs, Neocallimastigomycota have been found to be responsible for the fermentation of 18–63% of untreated plant biomass [56, 84, 85] despite only being approximately 8% of the gut microbiome biomass [86]. In bovines, the removal of anaerobic fungi through treatment with cycloheximide and tetronasin was shown to reduce intake of low quality feed to 70% [87]. This is likely as the mechanical disruption and subsequent enzymatic break down by anaerobic fungi would ordinarily speed up feed break down and allow more rapid clearance of digesta [22, 87], underlining their important role in the digestion of crude lignocellulose and subsequently host nutrition.

**Table 1:**
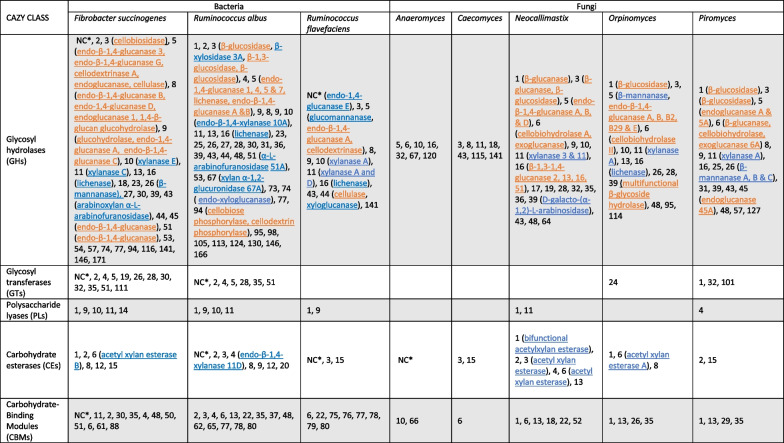
CAZyme families from prominent fibre degrading microbes isolated from the equine gut

Numbers under taxa indicate CAZyme families found within the corresponding CAZy class in each taxon. Fungal CAZymes found are heterologously expressed within each genus. Where enzyme functionality has been characterized, the protein name is written in brackets after the CAZy family number. Fibrolytic activities in blue are involved in hemicellulose degradation, while activities in orange are primarily cellulose degrading. Note- this is not an exhaustive list of fibrolytic microbes found in the equine gut, but rather a summary of those for whom enzymology has been described

*NC = CAZymes “non classified” CAZymes; i.e. not yet assigned to a family

In addition to their enzymatic potential, anaerobic fungi offer an added benefit in fibrolysis through mechanical agitation and hyphal invasion of plant tissue [59]. Helium ion micrographs of the equine derived anaerobic fungi *P. finnis* presented by Lillington et al. [59], demonstrated the dynamic mechanical and enzymatic break down of plants by anaerobic fungi including capturing of surface localised cellulosomes. Images revealed fungal rhizoids invasively covering grass particles, enhancing surface coverage and access to carbon sources. Also visible was hyphal penetration of plant substrate to access trapped carbon [59]. Hyphal tips, furthermore, have a high concentration of fibrolytic enzymes, the activity of which increase nutrient availability for other cellulolytic microbes including bacteria [62].

The simple monomers from lignocellulose degradation, such as glucose, are metabolised by fungi though a type of mixed acid fermentation (i.e., heterofermentative processes), where degradation of carbohydrates leads to the production of hydrogen, carbon dioxide, formate, acetate, succinate, and ethanol as by-products [21] (see Fig. 2 for an overview of anaerobic fungi plant break down and metabolism). The bulk of fungal metabolism is conducted by hydrogenosomes, an evolved version of the membrane bound organelle mitochondria used by respiration dependent organisms [21, 88, 89]. Rather than carrying out oxidative phosphorylation, this structure produces ATP through substrate-level phosphorylation under anoxic conditions, producing hydrogen as an end-product [21]. This hydrogen is considered vital for the growth of methanogenic archaea and bacteria [21], highlighting the importance of anaerobic fungi in the equine GIT microbial ecosystem.

Donkeys have been demonstrated to have increased fibre degradation abilities compared to horses [90] which is possibly explained by a study [73] comparing the microbial populations of different Equidae species, which found donkeys to have a six-fold greater anaerobic fungal loads compared to horses, as determined using quantitative polymerase chain reaction methods. Another key finding from that research was the strong correlation of diet with the types and concentrations of anaerobic fungi identified. The composition of the mycobiome was found to have a higher association with diet than species types, (e.g., zebra, horse, donkey) [73], highlighting the importance of equine nutrition in mycobiome development.

Past and present investigations into the equine hindgut have revealed its distinction from other anaerobic fungi ecosystems, both functionally and taxonomically. Several studies have revealed the presence and dominance of the genera *Khoyollomyces* (formally known as AL1), in the equine hindgut, having been found almost exclusively in equines [39, 44, 53, 55]. The type species *Khoyollomyces ramosus* (khyollo meaning horse, myces meaning fungi) was initially cultivated from zebra and equine faeces [68]. Isolates of *K. ramosus* were observed to produce monoflagellated zoospores and develop highly branched rhizoids when encysted [68]. The extensive rhizoidal branching of this species may explain its increased disruption of plant tissue compared to other anaerobic fungal species [59, 83], however this is yet to be investigated. The recent cultivation and, therefore, characterization of this genus explains why its role in the equine gut, and potentially novel enzymatic and metabolic pathways, are currently unknown.

The adaptation of anaerobic fungi to the gut environment of horses has been further emphasised through metabolic studies into equine derived fungi. Perhaps the most well documented genera of anaerobic fungi in herbivorous mammals is *Piromyces *spp.*,* including their production of highly effective cellulolytic enzymes. Interestingly, metabolic differences have been reported between strains of *Piromyces* isolated from equid and rumen guts, with equine derived strains possessing higher fibre degrading capabilities and overall faster growth rates [91]. After 25 h of growth on soluble sugars, fungal biomass (mg/ mL) from equine derived strains (ponies and donkeys) was three-fold higher than that of rumen strains grown on the same substrate. Digesta spends a shorter amount of time in the equine caecum compared to the rumen foregut, and therefore equine-strains of *Piromyces* have likely adapted to induce plant cell wall break down at an increased rate [91]. Of particular significance within the *Piromyces* genus is *P. equi*, a fungus isolated from equines that was found through protein and enzyme assays in conjunction with matrix-assisted laser desorption/ionization time-of-flight mass spectrometry analysis, to possess a major exoglucanase- Cel6A. Cel6A is one of the most widely studied cellulolytic enzymes as it is capable of fully digesting cellulose [92, 93], a key example of the powerful fibrolytic enzymes at play in equine digestion.

Several uncultivated anaerobic fungal clades have also been identified in the equine gut, including NG1, NG2, NG3, NG5, NG7, DT1, KF1, SK1 and SK3 [73, 74, 80]. However, many limitations currently exist in both traditional and molecular identification of anaerobic fungi, including their stringent cultivation requirements, A-T and repeat rich genome, unknown ploidy, lack of a reliable DNA barcode and complex physiology [94]. Additionally, reference sequences for these taxa are not well represented in publicly available databases for both their taxonomy and functionality. Tackling these technical issues is essential to improving our understanding of the role these microbes play in fibrolysis.

### Thermophilic fungi

Only recently has it been demonstrated that oxidative cleavage of cellulose occurs in the equine gut via aerobic thermophilic fungi [95]. Thermophiles are a type of extremophile microbe that can survive relatively high temperatures, over 100 °C for some Eubacterial and methanogenic Archean species [96, 97], or up to 60 °C for thermophilic fungi, the only eukaryotes demonstrated to grow at such high temperatures [98]. Thermophilic fungi, similar to anaerobic fungi, are a point of interest due to their rapid growth rates and high cellulolytic activity and have been isolated from herbivore faeces on several occasions, including from the horse [99, 100]. However, it was initially thought that the fungi colonized faecal content post defecation and consequently thrived in the warm conditions.

Studies that uncovered this process [98, 99] noted that gut dwelling anaerobic fungi seemingly lack auxiliary activity 9 family of enzymes (“AA9”) including lytic polysaccharide monooxygenases. These enzymes cleave cellulose fibres via C1 and C4 oxidation [101], making fibres more readily digestible by other microbial groups. The study successfully isolated C1 and C4- oxidized cellulose from both horse faeces and directly from the horse stomach and subsequently isolated three thermophilic fungal species which were cultivated at 50 °C; *Chaetomium thermophilum*, *Thermoascus aurantiacus* and *Scytalidium thermophilum* [95]*.* All three species when isolated directly from equine digesta were found to express AA9 enzymes, confirming their role in cellulose cleavage within the equine stomach. The study went on to postulate that these aerobic fungi grow in the anterior region of the horse gut where oxygen is consumed, consequently facilitating the anaerobic conditions of the lower gut [95]. Notably, enzyme activity of thermophilic organisms is often maximised at temperatures higher than that of the equine gut, and as such, it remains to be investigated how actively involved they are in plant break down within the equine GIT.

### Facultatively aerobic yeasts

The role and presence of yeasts in the equine hindgut is even less explored than anerobic fungi, which are rarely reported as residents of this ecosystem. All yeasts belong to two phyla; Ascomycota and Basidiomycota within the Dikarya subkingdom [102]. Yeasts are highly ubiquitous in the environment, found in a variety of habitats as pathogens, transients, or symbionts [103, 104]. Although some yeast species enter the equine gut, they are often considered non-functional transients in this eco-system, despite their ability to survive in this niche environment [105]. Yeasts found within herbivore digestive tracts are not strictly anaerobic like their mould and bacterial counterparts but instead considered ‘facultatively aerobic’, being able to survive with little to no oxygen [105]. This is due to mitochondrial adaptations in the form of deletions, mutations or duplications of mitochondrial DNA or nuclear DNA involved in oxidative phosphorylation [106]. These species, often referred to in the literature as ‘petites’, are respiration deficient but viable, and unable to grow on non-fermentable substrates such as ethanol or glycerol [106].

Much research on yeasts in herbivore digestion has been in insects, however the insect hindgut is more adapted to re-absorption of amino acids rather than plant break down, and there is otherwise little overlap between the GIT of these and horses. Some yeasts found in the aforementioned studies include genera also isolated from the equine gut, including some *Candida* and *Saccharomyces* species [105, 107]. Yeasts isolated from the gut of insect herbivores, namely termites and beetles, have been shown to produce extracellular enzymes, particularly xylan- and arabinogalactan-hydrolysing glycosidases, to break down hemicellulose and detoxify toxins in the herbivore diet [108–110]. These findings, although not demonstrated in equine derived yeasts, allude to the role of GIT yeasts in contributing to plant break down in conjunction with their other microbial counterparts.

## Fibrolytic bacteria

Bacteria are the most well documented microbial group within the equine GIT ecosystem, and undoubtably have important roles in fibre degradation and host health. While the cellulase diversity of anaerobic fungi is greater, cellulolytic bacteria are often found in higher concentrations throughout the GIT and have faster growth rates, consequently often being found to have higher cellulase counts [111]. Gut dwelling bacteria are well studied in mammalian hosts, having several proven roles in immunoregulation and other cell regulatory mechanisms [112]. Their fibrolytic role has been well established in the rumen caecum [113–123] and to a lesser extent, the equine hindgut [3–8].

The functionality of gut dwelling bacteria can broadly be classified into proteolytic, lactate-using, glycolytic and cellulolytic bacteria, the latter of which are mainly composed of species from the phyla Actinobacteria, Firmicutes, Proteobacteria and Bacteroidetes [12, 124]. Few cellulolytic bacteria have been isolated from the GIT of equines through culture dependent methods [25], possibly due to their slow growth rate and purported long lag phase to initiate substrate digestion [125]. Additionally, it is estimated that 33–80% of bacteria in the gut of herbivores are oxygen sensitive, including most cellulolytic bacteria [9] further challenging attempts to culture them and conduct functional evaluation. Current estimations of cellulolytic and hemicellulolytic bacterial populations in the equine hindgut range from 10^4^ to 10^8^ cells/ mL and 10^6^ to 10^8^ cells/ mL respectively, showing their dynamic functionality in breaking down different plant wall components within the hindgut [126].

Few studies have undertaken enzymatic profiling of equine fibrolytic bacteria. Genome annotation is a common method for evaluating the enzyme richness of microbial species, however, it is especially difficult for environmental and otherwise complex samples, due to the large diversified amount of data yielded from these analyses, and general absence of environmental species in current databases [127–130]. Through shotgun metagenomic sequencing, Gilory et al. [110], recovered 123 metagenome assembled genomes (MAGs) belonging to archaea and bacteria from five horse faecal samples. The bacteria detected were predominantly members of the phyla Proteobacteria, Firmicutes, Bacteroidetes and Actinobacteria, and collectively possessed a diverse array of polymer degrading enzymes [111]. Each bacterial MAG possessed on average 69 different CAZymes with Bacteroidota phyla members having the largest CAZyme repertoire. The majority of reported CAZymes belonged to the glycosyl hydrolase family (51%), indicating the potent role of these microbes in the break down of complex carbohydrates [111].

The enzymatic profiles of different microbial groups within the equine gut typically shows trends of anaerobic fungi being the primary degraders of cellulose, while fibrolytic bacteria target the degradation of hemicellulose. This functional specificity has particularly been demonstrated in the rumen. One such study assessed kingdom specific functionalities within the rumen microbiome through placing nylan bags containing switch grass directly into the rumen of two fistulated cows [131]. Metatranscriptomics on the rumen incubated bags revealed bacterial populations were primarily responsible for hemicellulose degradation, with the proteome of *Fibrobacter succinogenes* in particular containing a wealth of CAZymes from hemicellulose prominent families (GH11, GH51 and GH94). The proteome and transcriptome of rumen fungi however appeared better equipped for the degradation of cellulose structures with enzymes from the glycoside hydrolase family GH48 being the most abundant CAZymes detected [131].

The enhanced hemicellulolytic catabolism of equine caecal bacteria has been further demonstrated through their substantial growth on xylan rich media, the main carbohydrate component of hemicellulose, and subsequent poor colonization on cellulose rich media [6]. The xylanase activity of the dominant horse caecal isolate, *Enterococcus casseliflavus*, was also superior to that of isolates from buffalo and horse dung. The same study also found growth of an unidentified ligninolytic bacterium on lignin. Other cellulolytic bacteria isolated from horse faeces with substantial hydrolytic capacity include *Paenibacillus polymyxa*, *Enterobacter cloacae* and *Escherichia coli*, the former of which yielded the highest cellulolytic activity of those isolated [7].

Firmicutes are the largest phylum represented in the equine GIT, ranging from 40 to 90% in different sections [132]. Within this phylum is the class Clostridia which contains the obligately anaerobic family *Lachnospiraceae*. *Lachnospiraceae* are regarded as core microbiome members across most animals [133–135], and are recognised for their ability to degrade a variety of plant polysaccharides [136], resulting in the production of short chain fatty acids such as butyrate which has a demonstrated protective effect on colonocytes and serves as their main energy source [137]. The families *Ruminococcaceae* and *Fibrobacteraceae* are also members of Clostridia, and while often only representing a small portion of the equine microbiome, have been found as core members in the equine gut [9, 138]. *Ruminococcus* and *Fibrobacter* are the two main genera involved in cellulose degradation in the equine gut, specifically, *Ruminococcus albus*, *Ruminococcus flavefaciens* and *Fibrobacter succinogenes*, and which are often used as markers for cellulolytic activity in herbivore gut studies [23] (see Table 1 for an overview of functional enzymes).

Ruminococci are one of few bacteria isolated from the gut with the ability to produce cellulosomes [139, 140]. As previously mentioned, the use of functionally organizing CAZymes into a cellulosomal network greatly enhances the fibrolytic capacity of an organism. *R. flavefaciens* has frequently been identified as the main cellulolytic bacterial species in the equine gut, ranging in concentrations throughout the lower GIT of between 2 and 9.7% of overall bacterial populations [3, 141]. Assembly of a cellulosomal complex from *R. flavefaciens* revealed a diversity of CAZyme family domains, including glycosyl hydrolase, carbohydrate esterase and pectate lyase binding domains, however the function of around 30% of these proteins remained unknown [140]. A unique feature of the cellulosome of *R. flavefaciens* is its adaptor scaffoldin, ‘ScaC’, which possesses the ability to bind to different dockerin groups and consequently modulate enzyme integration into the cellulosome complex based on functional needs [142]. Furthermore, the cellulosome of *R. flavefaciens* showed preferential recruitment and appendage to hemicellulases, highlighting the prominent role of bacteria in hemicellulose degradation [142].

The ability of bacterial cellulosomes networks to tightly adhere to plant fibres within the gut has been recently revealed [143], further enhancing microbe-substrate interactions. *Ruminococcus champanellensis*, a cellulolytic bacterium isolated from the human gut, employs cellulosomes as a mechanism to anchor itself to plant substrates and withstand the motility of the GIT [143]. Through modelling and molecular dynamic simulations, researchers were able to show the cohesion-dockerin complex of these cellulosomes were able to regulate the adhesion of bacteria to substrates under different environmental conditions such as pH and high stress, with the protein bond becoming stronger as force increased [143]. No studies have yet reported the isolation of this *Ruminoccous* species from the equine gut, however the presence of close relatives and other cellulosomal producing species suggests this is a mechanism likely employed by equine cellulolytic bacteria as well [144].

Several studies looking into the occurrence of equine metabolic disorders analysed fluctuations on cellulolytic bacteria under different conditions [8, 38, 39]. A case study on fifteen horses treated with different antibiotics, showed a remarkable decrease in the presence of cellulolytic bacteria (> 99% decrease), following treatment, through culture-based methods [8]. After antibiotic withdrawal, the levels of cellulolytic bacteria in treated horses remained substantially lower than in control horses, and continued to decrease for a week after withdrawal [8]. Subsequently, this decrease in beneficial GIT bacteria allowed colonization of potentially pathogenic bacteria, including *Salmonella* and *Clostridium difficile* which are common causes of diarrhea and other equine GIT disorders [8]. A decrease in cellulolytic bacteria in the equine large intestine was shown to correspond with a decrease in the production of acetate, indicative of decreased fibre degradation [38].

## Microbial synergism

Microbial synergism is defined as the mutually beneficial increase in the productivity or growth of a microbial community resulting from the metabolic interactions of two or more microorganisms, to which their combined effect is greater than the sum of their separate abilities [145, 146]. This is generally achieved through nutritional interdependence, whereby one species can utilize the products of another, minimizing by-products of the producer while providing nutrition to the feeder [147, 148].

Fungi and bacteria both have independent, but synergistic, fibre degrading roles in the equine hindgut, enhanced by their interactions with one another and other microbes present. As previously described, anaerobic fungi are the primary colonizers of plant biomass within the equine gut, their mechanical disruption of plant cuticles [83] enabling fibrolytic bacteria access to plant fibre which they would not otherwise be able to ferment [33]. The positive effects of anaerobic fungi on bacterial populations have particularly been demonstrated through administration of anaerobic fungal cultures as feed additives. Paul et al. [149], fed cultures of the anaerobic fungus *Piromyces* sp. FNG5 (previously isolated from wild bull faeces) to buffaloes and showed significant increases in cellulolytic and hemicellulolytic bacterial counts, as well as increases in resident fungal populations [149]. Increases in fibrolytic microbial populations was concomitant with increased volatile fatty acid production and increased xylanase, cellulase, protease, and acetyl and feruloyl esterase activities [150]. This is consistent with other studies demonstrating that the addition of fibrolytic fungi, to animal feed, increases bacterial populations two-fold and subsequently enzyme activities and feed digestibility within the gut [151, 152].

Yeast supplementation has been shown to have beneficial effects on rumen bacterial populations through increased production of short chain fatty acids and vitamins [153, 154], however results in equines have been less consistent. Garber, Hastie and Murray [16] have compiled a list of studies that use the yeast *Saccharomyces cerevisiae* as a probiotic in equines. Of five studies conducted, two recorded little to no effect on bacterial populations analysed from either the gut or faeces [155, 156], and the other three noted variable effects [103–105]. In response to yeast supplementation, one study noted decreased *Streptococci* in the colon and increased *Lactobacillus* in the caecum [157], while another study recorded overall decreased *Lactobacilli* counts in faeces [158]. The fifth study recorded reduced *F. succinogenes* populations in faeces but otherwise no effect on bacterial populations or equine fibre digestibility [159]. Ultimately it remains unclear whether yeasts in the equine GIT can produce substantial changes in the digestibility of plant substrates. A deeper understanding of the role of yeasts within the equine gut would greatly facilitate development of a more suitable feed supplement to assist plant matter break down.

The symbiotic relationship between anaerobic fungi and methanogenic archaea has been a point of interest in herbivore digestive systems [160], however little work has been done on understanding this relationship within the equine gut, with archaea only being first isolated from the equine gut in 1996 [161]. The diversity of methanogenic archaea within the equine gut varies along the GIT and depending on host animal, with the horse hindgut showing a predominance for *Methanobacterium*-like sequences, and the donkey hindgut housing more *Methanocorpusculum*-like sequences [162]. The production of methane by methanogenic archaea within the herbivore gut is a by-product of fibre digestion [163, 164]. Hydrogen is produced by anaerobic fungi and bacteria and is reduced to methane by methanogens, allowing maintenance of low hydrogen levels within the gut, to consequently maintain thermodynamic requirements for anaerobic fermentation [165].

In addition to the beneficial waste removal by methanogenic archaea, their interactions with anaerobic fungi have also demonstrated increased transcription of important CAZymes, ultimately enhancing fibrolytic capacity [166]. Co-cultivation of the bulbous anaerobic fungi *Caecomyces churrovis* and the methanogen *Methanobacterium bryantii* resulted in a stable culture which was then evaluated metabolically and transcriptionally [166]. Genome analysis found that almost 1% of all adenines were methylated, with at least 6% of the methylated genes belonging to CAZymes [166]. DNA methylation is an epigenetic process in which methyl groups are added to a DNA molecule, which can change the activity of a DNA segment through state specific control of gene expression [167]. Furthermore, co-culturing of the anaerobic fungi and methanogen resulted in a proportional increase of CAZyme gene expression, the majority of which were either carbohydrate binding modules, fungal dockerin domains or of unknown function [166]. Some cellulases contain an active domain site for binding to carbohydrate binding modules [168], with those bound to a carbohydrate binding module consistently demonstrating enhanced cellulose attachment [169–171]. Carbohydrate binding modules have diverse ligand specificity and can maintain enzyme–substrate proximity, ultimately leading to prolonged and steady enzyme–substrate interactions and increased enzyme concentrations on the polysaccharide surface [169, 172]. These enhanced interactions can increase the hydrolytic activity of enzymes on soluble substrates [173], however deeper investigation is needed on their role in plant break down within an intestinal setting.

## Future directions: towards developing a functional assay

Understanding the composition and functionality of fibrolytic microbial communities within the equine hindgut is of great importance in equine nutrition and to optimise energy yield from plant matter. Gastrointestinal disturbances induced by changes in the gastric microbiome (‘dysbioses’), brought on by disease, change of diet, antibiotic use, age or other factors, can result in compositional shifts of the equine microbiome, resulting in fermentation dysfunction and ultimately metabolic disorders [174]. In the instance of the equine gut, identifying microbes present within this environment and the repertoire of enzymes they possess can help researchers and veterinarians alike better understand the taxa associated with a healthy or diseased animal, and on a larger scale, can help researchers underpin the important taxa involved in complex fibre degradation.

It is evident through elucidation of the fibrolytic capacity of the equine gut that more detailed investigations of this community require a holistic understanding of both taxonomy and functionality, with an emphasis on enzymology of particular importance in crucial functions involved in fibre break down. While taxonomically identifying bacteria and fungi from complex microbial communities has made significant headway in recent years, there are still several drawbacks that limit its usability in fully unravelling the complexity of ecosystems like the equine gut. Kauter et al. [132] provided an overview of current taxonomic methods used for analysing this microenvironment, and further evaluated the sequencing technologies available. Taxonomically, some of the major issues hindering identification of this community include, database limitations [128], substantial size polymorphism for anaerobic fungal DNA barcodes (up to 13% polymorphism between clones of a single *Piromyces* strain [175]) and low intra-species taxonomic resolution when using short read sequencing [73]. Identifying important functions and their corresponding genes within a mixed community has the potential to improve our understanding of the break down of complex polysaccharides in plant material within the equine gut and the specific genes involved in these processes.

In recent years, shotgun metagenomics has provided a wealth of insight into the functionality of complex microbial communities, highlighting the varying functional roles of different taxa and how they work together independently and synergistically [176]. Peng et al. [176], recently profiled the bacterial and fungal communities from goat faeces, targeting the taxa and enzymes responsible for lignocellulose break down. Briefly, faecal pellets were cultured anaerobically on a range of enrichment substrate media (alfalfa stems, canary grass, xylan or sugarcane bagasse) with varying antibiotic treatments to bias the growth of anaerobic fungi alone, or anaerobic fungi and methanogenic archaea. Shotgun metagenomics on samples yielded 18 eukaryotic derived MAGs, the annotated CAZymes which were categorized into either cellulose,- hemicellulose-, or pectin/ ester-degrading. The total number of lignocellulose-active genes from eukaryotic derived MAGs was significantly higher than that of prokaryotic derived MAGs, with a number being found only in fungi, indicating the incredible untapped potential of anaerobic fungal enzymes [176]. Through co-culturing microbial groups on different media and analysing metabolic outputs, the study was also able to assess the functional performance of different microbial consortia in degrading lignocellulose. In line with previous studies, it was found that the fungi- and archaea-dominated community not only degraded several substrates better than bacteria-dominated consortia, but also had significantly higher levels of cellulosomal CAZymes compared to the bacterial consortia.

Large scale phenotypic assays are another valuable method in uncovering key taxa involved in important targeted functions, such as fibre degradation. Recently, 1031 fungal strains acquired from a diversity of habitats were used in a large-scale multi-phenotyping assay to determine their ability to degrade non-natural industrial compounds such as plastics and dyes [177]. This assay involved culturing each strain on six different media with different degradation-resistant compounds, to determine their usability as industrial biocatalysts. Their ability to grow on these media and utilise the given substrates was determined by evaluating different degradation phenotypes depending on the substrate being utilized including change of media colour, oxidization, fungal growth, and halo clearing. The study demonstrated significant functional diversity within taxonomic ranks, with all families having at least one strain with high degradation abilities. Such findings highlight the importance of identifying fungal isolates to low taxonomic ranks (genera and species level) to fully evaluate the role of the taxa present. A focus on enzymology rather than taxonomy may also allow the elucidation of rare taxa involved in fibre break down through production of prominent CAZymes involved in fibre degradation.

Explorative microarrays, such as the ‘FibroChip’, are emerging molecular tools that utilise transcriptomics to detect genes and their genetic variants from mixed community samples [178]. Such tools can rapidly and relatively inexpensively monitor in parallel the functional potential of a high number of samples, with current analyses proving easier and quicker than other metatranscriptomics and lengthy gene annotation studies [178]. The FibroChip microarray was composed of thousands of probes targeting hundreds of CAZyme genes from eight families that were believed to have important and complementary roles in cellulose and hemicellulose degradation [GH5, 9, 10, 11, 43 and 48 and CE1 and 6]. The study was ultimately able to validate the ability of the FibroChip to detect differential gene expression in bacteria and was even able to identify differential gene expression between clone isolates cultivated on different substrates. The FibroChip was further used to analyse total RNA isolated from the rumen fluid of a cannulated cow, and while there were gaps in the enzymology of fungi, ultimately proved a valuable stepping stone towards simultaneously taxonomically and functionally analysing complex fibrolytic communities [178].

With the evolving use of long read sequencing platforms such as Nanopore Sequencing from Oxford Nanopore Technologies and single-molecule-real-time sequencing (SMRT) from Pacific Biosciences, the speed and accuracy at which we can screen genetic material from microbial communities for enzymatic genes and taxonomic barcodes is continuing to improve [179, 180]. At the moment, both technologies can rapidly generate millions of full-length reference sequences (over 10 kb) within a couple of hours [181], however come at the cost of increased error rates compared to more traditional sequencing platforms [182]. SMRT sequencing has been used to build fungal genomes for functional annotation, unveiling novel fungal genera from the equine gut [68] and elucidating the complexities of the cellulosomal complex from *P. finnis* [81]*.* The application of these technologies in large scale enzyme screening has not yet been undertaken, however with continued research would likely prove an invaluable tool in the simultaneous taxonomic and functional assessment of complex microbial ecosystems, in conjunction with traditional phenotypic assays.

## Conclusion

The equine gut houses a range of unique fungi, bacteria, archaea, and other microbial groups which act synergistically to break down complex plant fibres that enter the digestive system. The interdependent and symbiotic interactions occurring within the equine gut, including sequential metabolic handoffs and epigenetic alterations of functional gene expression, mark this microbial community as both incredibly complex and powerful in its fibrolytic capacity. Understanding the microbes present within this ecosystem, particularly anaerobic fungi and cellulolytic bacteria, and their functional genes, would have widespread industrial, veterinary and research applications.

## Data Availability

Not applicable.

## Abbreviations

DNA: Deoxyribonucleic acid
GIT: Gastrointestinal tract
SMRT: Single-molecule-real-time sequencing
Kb: Kilobases
MAG: Metagenome assembled genome
